# Predicting eye movements in a contour detection task

**DOI:** 10.1186/1471-2202-13-S1-O4

**Published:** 2012-07-16

**Authors:** Udo Ernst, Humbeeck Van Nathalie, Nadine Schmitt, Frouke Hermens, Johan Wagemans

**Affiliations:** 1 Institute for Theoretical Physics, University of Bremen, Bremen, Germany; 2 Laboratory of Experimental Psychology, University of Leuven, Leuven, Belgium

## 

An important task for the visual system is grouping local image elements into meaningful objects. One fundamental process for performing this task is contour integration, in which collinearly aligned local edges are merged into global contours. Models for contour integration often use iterative algorithms to explain how this cognitive process is performed in the brain. By employing an association field (AF) which quantifies how strongly two oriented edge elements are linked to be part of a contour, such a model integrates edge elements in a recurrent manner. This process generates saliency maps for contours of increasing lengths as time proceeds.

Recently, we developed a probabilistic model of contour integration which explains human contour detection behavior to a previously unprecedented degree [1]. Given this performance, we wondered whether the model might also explain the spatiotemporal dynamics of contour integration. Measuring eye movements can be a useful method to test the corresponding model predictions, hypothesizing that subsequent fixations of subjects preferentially visit ‘hotspots’ of neural activity which dynamically emerge during the integration process.

Here we compare model simulations with data from a recent experiment [3], in which eye movements were measured while observers were instructed to search for a 7-element contour embedded in a background of randomly oriented Gabor elements [2]. The experiment consisted of two tasks: for the first task observers were asked to indicate whether a global contour was on the left or right hemifield (left-right task), while the second task required observers to indicate presence or absence of a contour (present-absent task). The parameters of the model were first optimized for the left-right task, requiring it to reproduce both human performance and decisions as best as possible.

**Figure 1:**
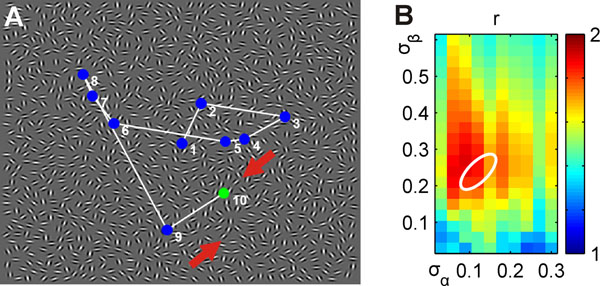
**A.** Sample stimulus containing a contour (red arrows), with overlaid saccade trajectory of one subject. **B.** Factor *r* by which model activity in the second task is higher at saccade target locations than at other locations, in dependence on two parameters defining the shape of its association field. The white ellipse denotes the parameter regime for which the model optimally fitted human contour detection behavior in the first task.

The optimal model was then used to predict potential locations for saccade targets which we compared to fixation trajectories of observers for stimuli from the second task in which no contour was present. For edge elements near saccade targets, the model predicts a probability to belong to a contour which is two times higher than for other edge elements. Thus, the statistical analysis shows that fixations are indeed not random, but are likely to occur on locations judged salient by the model. This result confirms both the validity of our model and the hypothesis that saccades on random Gabor fields preferentially visit locations with edge configurations similar to contours.

## References

[B1] Ernst UA Mandon S Schinkel-Bielefeld N Neitzel SD Kreiter AK Pawelzik KR Optimality of human contour integration PLoS Comp Biolin review 10.1371/journal.pcbi.1002520 PMC3360074 22654653

[B2] Field DJ Hayes A Hess RF Contour integration by the human visual system: Evidence for a local “association field.” Vision Research 1993 33 173 193 10.1016/0042-6989(93)90156-Q 8447091

[B3] Van Humbeeck N Hermens F Wagemans J Eye movement strategies during contour integration Perception 2011 40 ECVP Abstract Supplement 192

